# The gut microbiota–brain axis in neurological disorders

**DOI:** 10.1002/mco2.656

**Published:** 2024-07-20

**Authors:** Mingming You, Nan Chen, Yuanyuan Yang, Lingjun Cheng, Hongzhang He, Yanhua Cai, Yating Liu, Haiyue Liu, Guolin Hong

**Affiliations:** ^1^ Xiamen Key Laboratory of Genetic Testing The Department of Laboratory Medicine The First Affiliated Hospital of Xiamen University, School of Medicine, Xiamen University Xiamen China; ^2^ Master of Public Health School of Public Health Xiamen University Xiamen China

**Keywords:** Alzheimer's disease, autism, fecal microbiota transplantation, gut microbiota–brain axis, neurological disorders, Parkinson's disease, probiotics

## Abstract

Previous studies have shown a bidirectional communication between human gut microbiota and the brain, known as the microbiota–gut–brain axis (MGBA). The MGBA influences the host's nervous system development, emotional regulation, and cognitive function through neurotransmitters, immune modulation, and metabolic pathways. Factors like diet, lifestyle, genetics, and environment shape the gut microbiota composition together. Most research have explored how gut microbiota regulates host physiology and its potential in preventing and treating neurological disorders. However, the individual heterogeneity of gut microbiota, strains playing a dominant role in neurological diseases, and the interactions of these microbial metabolites with the central/peripheral nervous systems still need exploration. This review summarizes the potential role of gut microbiota in driving neurodevelopmental disorders (autism spectrum disorder and attention deficit/hyperactivity disorder), neurodegenerative diseases (Alzheimer's and Parkinson's disease), and mood disorders (anxiety and depression) in recent years and discusses the current clinical and preclinical gut microbe‐based interventions, including dietary intervention, probiotics, prebiotics, and fecal microbiota transplantation. It also puts forward the current insufficient research on gut microbiota in neurological disorders and provides a framework for further research on neurological disorders.

## Graphical abstract

Gut microbiota driving neurological disorders. Gut microbiota may drive neurodevelopmental disorders (ASD and ADHD), neurodegenerative disorders (AD and PD), and mood disorders (anxiety and depression) through gastrointestinal disorders, gut microbiota disorders, decreased diversity of gut microbiota, delayed development of gut microbiota, and so on.

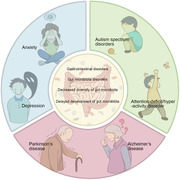

## 1 INTRODUCTION

Microbes are the oldest life on Earth and have coevolved with their human hosts for millions of years. In the past few decades, accelerated research on the microbiome has revealed that microbiota is a key determinant of human health and disease, maintaining host homeostasis and regulating host physiological state. At the genetic level, 99% of the genes in the human body are microbial genes, and there is unique microbiome colonization in human skin, respiratory tract, urogenital tract, gastrointestinal tract (GI), and so on.^1^, ^2^ Among them, the human GI is the host of most microbiota; there are trillions of microbes, and over time, they coevolve with the host organisms and depend on each other.^1^ These microbial communities present in the human gut are called the gut microbiome. In the course of the lifespan, the gut microbiota exhibits dynamic fluctuations influenced by factors such as age, psychological disposition, lifestyle, and GI health status of the host. This dynamic and diverse microbial community actively shapes and modulates the host's pathophysiological processes, thereby underscoring its significant potential for clinical diagnosis and therapeutic intervention.^3^, ^4^, ^5^, ^6^ It also promotes the human microbiome project that is put forward, in order to understand our microbial components in genetic and metabolic landscape.^7^ With the progress of research on the intersection of gut microbiota and neuroscience, some evidence has clarified the existence of bidirectional communication pathways between gut microbiota and the central nervous system (CNS), which affect the synthesis and release of neurotransmitters in the brain. These studies highlight the close association between gut health and neural health, suggesting a potential impact on neurodevelopmental disorders.^1^, ^8^ The biphasic communication between gut microbes and the CNS is known as the “microbiota–gut–brain axis” (MGBA).^9^ Current cellular models, mouse models, and human experimental studies consistently demonstrate that the gut microbiome plays a critical role in various aspects of nervous system development, neuroinflammation, cognitive processes, emotion regulation, and behavioral regulation.^10^, ^11^, ^12^ Therefore, the gut microbiota is a personalized, multifunctional target with great potential in the diagnosis and treatment of neurological disorders.

The purpose of this review is to elaborate on the functional role of the MGBA and the current research progress in neurological disorders (such as autism spectrum disorders [ASD], attention‐deficit/hyperactivity disorder [ADHD], Alzheimer's disease [AD], Parkinson's disease [PD], depression, and anxiety), so as to provide a summary framework for this rapidly developing research field.

We have summarized the following key points regarding the role of MBGA in several neurological disorders described here: (1) it has been reported that the gut microbiota of patients with neurological disorders is different from that of healthy individuals, and some even have the common characteristic of gut microbiota disorder; (2) depletion or delayed development of gut microbes may cause neurodevelopmental disorders, and higher inflammatory markers are both associated with a decrease in beneficial bacteria and an increase in proinflammatory bacteria; (3) neurodegenerative diseases are associated with decreased diversity of gut microbiota, and the deposition of some characteristic proteins is also regulated by gut microbiota; (4) GI disorders are seen as complications or prodromal symptoms in most neurological disorders, which also implies the role of MGBA in neurological disorders; (5) although some studies have shown beneficial results, the limitations and complexity of the intervention treatment of gut microbiota in the above neurological disorders by probiotics, fecal material transplant (FMT), diet, and other means need to be discussed and further studied.

This review explores the diversity of gut microbes and the factors influencing them (dietary, genetic, and environmental) and the bidirectional communication of the MGBA. It mainly summarizes the role of the MGBA in the pathogenesis and risk factors of neurological disorders, particularly based on human and animal models, including differences in microbiome and metabolites. Furthermore, the review delves into the modulation of gut microbes in clinical treatments (such as probiotics, prebiotics, fecal microbiota transplantation [FMT], and dietary interventions), discussing their potential benefits and limitations.

## 2 THE GUT MICROBIOTA

### 2.1 Composition and diversity of the gut microbiota

The gut microbiota is not only a large number but also a complex community with richness and diversity, including bacteria, archaea, fungi, viruses, parasites, and protozoa, among which bacteria are the most important components.^1^ Previous studies on human gut microbiome have shown that the majority of human gut bacteria are anaerobes, dominated by *Firmicutes* and *Bacteroidetes*, whereas *Actinobacteria*, *Clostridia*, *Cyanobacteria*, *Proteobacteria*, and *Verrucomicrobia* are less common.^13^ *Bifidobacteria*, a member of *Firmicutes*, are thought to play a role in the development of the immune system in early life and have anti‐inflammatory properties, which are considered to be beneficial bacteria.^14^, ^15^ The gut microbiota is diverse and has obvious individual differences.^16^ The dysbiosis of gut commensal microbiota and the increase of pathogenic microbiota can affect GI function, homeostasis, and overall health, thereby serving as pivotal contributors to disease pathogenesis.^2^, ^16^ In general, gut microbial diversity increases between childhood and adulthood and decreases with aging.^17^ The diversity of gut microbiota is a potential marker of healthy aging, and the alpha diversity of the gut microbiota decreases with age and is associated with frailty in the elderly.^18^ However, the diversity of gut microbiota is not reduced in all elderly people, and the diversity of gut microbiota is high in long‐lived people.^19^

Gut fungi and bacteria appear to exhibit a synergistic relationship in the occurrence of diseases.^20^, ^21^ *Candida*, *Saccharomyces*, and *Cladosporium* are the most abundant genera in the gut fungal flora of healthy adults.^20^ There are also a certain number of viruses in the gut, including bacteriophages and other viruses, and phage deployment is involved in the mechanism of colonization resistance.^17^ Phages can drive evolutionary changes in bacterial communities by creating gene flow networks that promote ecological adaptation.^22^ In addition, host assignment showed that virus diversity was highest in *Firmicutes*.^22^ The gut microbiome also contains methanogenic archaea (mainly *Methanobrevibacter smithii*).^16^ Overall, the structure and diversity of gut microbes need to be further revealed.

### 2.2 Factors influencing the gut microbiota

The composition of the human gut microbiota is subject to numerous influences. Initial colonization of gut microbes occurs during the early stages of human birth, with microbial transmission from the mother's birth canal, skin, and breast milk establishing the infant's nascent gut microbiota.^23^ After weaning, the development of infant gut microbiota enters a transitional stage, and enters a stable stage in childhood. Its composition, diversity, and function gradually resemble those of adults and become the original bacteria.^24^, ^25^ Some viewpoints suggest that the human microbiota may be exposed at earlier stages, potentially influenced by the placental microbiota during maternal pregnancy.^23^, ^26^ Subsequent studies have indicated that the placenta in healthy human pregnancies do not harbor microbes, suggesting that the detected “placental microbes” may be common contaminants.^27^, ^28^ The idea of the existence of “placental microbes” is currently controversial and needs further exploration.

Alterations in the structural diversity of the gut microbiota can modulate the physiological state of the host and potentially contribute to the occurrence of diseases.^17^, ^29^, ^30^, ^31^ Hence, it is crucial to understand the regulatory mechanisms governing gut microecology for effective disease prevention and treatment. Apart from the impact of birth stage on the initial establishment of human gut microbiota, various other factors contribute to its composition. In this context, we delineate the influence of dietary patterns, genetic predispositions, and environmental factors on the gut microbiome, with pertinent studies outlined in Table 1.

**TABLE 1:** Factors influencing the gut microbiota.

Factors	Diseases	Results	References
Diet			
Modified Mediterranean‐ketogenic diet (MMKD)	AD	Causes changes in gut bacteria and organic acids that correlate with AD cerebrospinal fluid (CSF) biomarkers	^32^
	MCI	The γ‐aminobutyric acid (GABA) is regulated in MCI patients.	^33^
Vegetarian diet	PD	PD‐associated microbiome changes occurred, Unified Parkinson's Disease Rating Scale Part III scores improved significantly, and levodopa equivalent daily dose decreased.	^34^
Mediterranean diet	PD	Roseburia abundance of PD patients is reduced, and constipation symptoms improve.	^35^
Processed food	ADHD	The processed food and ADHD groups showed significantly lower α‐diversity of gut microbiota than the control group.	^36^
Genetics			
	AD	The host genetic factors influencing the abundance of ten genera are significantly associated with AD.	^37^
	PD	Genetically increased Bifidobacterium levels correlated with decreased PD risk.	^38^
	ASD	The genera Prevotellaceae and Turicibacter predicted by genetic analysis may be positively associated with ASD. And genera Dorea, Ruminiclostridium5, Ruminococcus1 genera and Sutterella for ASD potentially protective role of the gut microbiota.	^39^
	Depression	Increased abundance of Morganella and Klebsiella has been causally associated with depression.	^40^
Environment			
Smoke	AD	Prevotella abundance was negatively correlated with smoking.	^41^
	PD	Smoking was associated with β‐diversity.	^42^
Geographic location	PD	Comparable findings to previous studies from the Northern hemisphere in regard to changes in gut microbiota composition	^43^
	ASD	The gut microbiome in individuals with ASD is affected by study‐site location as well as gastrointestinal symptom severity.	^44^
PM_10_ or NO_2_	ASD	PM_10_ and possibly NO_2_ exposure during early pregnancy may affect autistic traits at age 6 years through the alteration of Proteobacteria abundance.	^45^
As and Hg	ASD	Arsenic and mercury were significantly associated with Parabacteroides and Oscillospira in the gut.	^46^
Ethnicity	Depression	The gut microbiota is linked to depressive symptom levels and that this association generalizes across ethnic groups.	^47^

Abbreviations: CSF, cerebrospinal fluid; MCI, mild cognitive impairment; MMKD, modified Mediterranean‐ketogenic diet; γ‐aminobutyric acid, GABA.

#### 2.2.1 Diet

Diet is a key driver in shaping the gut microbiota. Dietary habits and types of food affect the microbial composition, which can be beneficial or harmful. There are significant differences in the composition of gut microbiota between people with different dietary habits. In early infancy, human milk oligosaccharides function as prebiotics, promote the growth and colonization of gut microbiota (mainly *Bifidobacterium* and *Lactobacillus*), and contribute to the shaping of the immune system in early life.^48^, ^49^ After weaning from breast milk, gut microbiota is further reshaped by long‐term diet. However, different dietary habits also have different effects on gut microbiota, and there are health‐promoting diets and disease‐related diets.

The Mediterranean diet (MD) is highly diverse and includes high amounts of fresh vegetables and fruits, legumes, whole grains, nuts, olive oil, fish, red wine, and a low intake of processed and red meat, characterized by a high level of dietary fiber. Dietary fiber intake can regulate the gut microbiota, improve gut microbial richness and biodiversity, and fermentation to produce short‐chain fatty acids (SCFA), such as propionate and butyrate.^50^, ^51^ Increased SCFA production by gut microbiota from dietary fiber can enhance glucose control and support a healthy metabolism in individuals with type 2 diabetes.^52^ The regulation of gut microbiota by MD is often manifested as the increase in the abundance of *Bacteroides*, *Bifidobacterium*, and *Prevotella*.^53^, ^54^, ^55^ A 1‐year MD intervention in older adults from five European countries showed that MD modulates the microbiome in ways that are negatively associated with frailty and inflammation and positively associated with health.^56^ Another cohort study of over 47,000 Swedish women showed that women who adhered to an MD pattern in midlife had a lower risk of developing PD in later life.^57^

The vegetarian diet is one that does not use any animal products. It is similar to the MD and includes foods rich in fiber, unsaturated fatty acids, and polyphenols. Compared with omnivores, vegetarians had a higher alpha diversity of gut microbiota, with more reported increases in *Prevotella* and *Bifidobacterium*, as well as decreases in *Firmicutes*.^58^, ^59^ The vegan diet is effective in promoting gut microbial diversity and preventing inflammation, which also helps lower cholesterol and body weight.^60^

Conversely, unhealthy dietary habits encompass industrialized foods and the Western diet, characterized by its high calorie content, abundant protein, and high fat levels. The Western diet has been linked to decreased diversity in gut microbiota, potentially elevating the risk of certain chronic conditions.^61^ Western diet can also alter specific microbiota communities, such as reducing anti‐inflammatory bacteria like *Bifidobacterium* and increasing the abundance of proinflammatory bacteria like *Firmicutes*.^62^, ^63^ This diet rich in fat and protein may affect gut microbial metabolites and promote the development of chronic inflammation and obesity.^62^, ^64^ A study involving animals demonstrated that prolonged lack of dietary fiber in mice can impact the synaptic structure of the hippocampus by changing their gut microbiota and is closely linked to cognitive impairments in mice.^65^ In a randomized controlled trial using the Western diet versus MD in patients with mild cognitive impairment (MCI), MD was observed to benefit AD cerebrospinal fluid (CSF) biomarkers and memory in cognitively normal adults, whereas the Western diet with high fat had negative effects.^66^

#### 2.2.2 Genetics

The role of genetic factors in determining the composition of the gut microbiome is significant. Microbiome genome‐wide association studies have identified correlations between individual host genetic sites and the gut microbiome, as well as the capacity of particular host metabolic molecules to influence the gut microbiome.^67^ For example, the strongest correlation was found between the LCT gene and the genus *Bifidobacterium*, PLD1 was associated with *Akkermansia*, and copy number of the amylase gene AMY1 was associated with increased *Prevotella*, *Porphyromonas*, and *Ruminococcaceae*.^67^, ^68^, ^69^, ^70^ The ABO gene is associated with *Bifidobacterium bifidum* and *Collinsella aerofaciens*.^70^ Research on the gut microbiota of twins in the UK revealed that monozygotic twins who had been separated for extended periods still exhibited a notable resemblance in gut microbial profiles when compared with dizygotic twins.^71^ *Christensenellaceae*, *Actinobacteria*, *Tenericutes*, and *Euryarchaeota* in *Firmicutes* were more heritable, whereas *Bacteroidetes* had very low heritability, and the dominant human gut archaeal *Methanobrevibacter* reached a significant level of heritability.^71^, ^72^ In another related study of host genetic–gut microbiome in an East Asian population, *Desulfovibrionaceae* and *Odoribacter* showed significant heritability.^73^

Furthermore, the pathophysiological state of the host could be linked to the interplay between host genetics and gut microbes. Mendelian randomization (MR) is a method of using genetic variants as instrumental variables to investigate the causal relationship between exposure and outcome in observational studies.^74^ Several MR analysis studies have suggested that higher abundance of *Actinobacteria* and their *Bifidobacterium* genus may be protective against ulcerative colitis, and higher abundance of *Oxalobacteriaceae* may be protective against rheumatoid arthritis.^68^ There was a suggestive association between genetically increased *Bifidobacteria* and lower risk for PD.^38^ Host gene‐driven increases in *Actinobacteria* at class level were suggestive of AD, whereas genetic increases in *Faecalibacterium* at genus level were associated with a protective effect against AD risk.^75^ However, of note, most of the microbial species that make up the gut microbiome appear to be unaffected by host genes and are mostly acquired due to diet and environmental exposures.^69^

#### 2.2.3 Environment

The composition of gut microbiota varies depending on geographic location, which can be affected by factors such as environmental pollution, water source and quality, socio‐demographic features, and cultural aspects associated with diverse lifestyles.^76^, ^77^, ^78^, ^79^

Many other studies have shown geographic or ethnic‐specific differences in gut microbiota.^76^ Host location has the strongest correlation with microbial variation among phenotypes, and microbiota‐based models of metabolic disease developed in one location do not appear to be generalizable to other locations.^80^ Moreover, the gut microbiota varies in the abundance of antibiotic resistance genes in the genomes of different geographical populations.^76^ Analysis of fecal microbes in infants at high risk for type 1 diabetes at six clinical centers showed that geographic origin was significantly correlated with the diversity and relative abundance of different bacterial genera, especially *Bifidobacterium*, *Veillonella*, *Faecalibacterium*, *Streptococcus*, and *Akkermansia*.^81^ A study of the gut microbiota of immigrants in the United States showed that the gut microbiota of immigrants converged to the Euro‐American microbiota after relocating to the United States.^82^ Increasingly, Western‐associated *Bacteroides* has replaced non‐Western‐associated *Prevotella* in the United States.^82^

As an environmental factor that cannot be ignored in industrialized cities, air pollution may also affect the composition of gut microbiota. Traffic‐related air pollution exposure was associated with the relative abundance of *Bacteroidaceae* and *Corynebacteriaceae*, which were associated with metabolic alterations and gut inflammation.^77^ Tobacco smoking reduces the abundance of Actinobacteria.^83^ Infants from smoking families had lower diversity and abundance of gut microbiota, especially *Bifidobacterium*, than those from nonsmoking families.^83^ In addition, the health of drinking water also plays a role in shaping gut microbiota. In mice study, the diversity of bacterial communities in mice drinking different types of water (autoclaved tap‐water, bottled mineral water, disinfected water, tap water) was significantly affected by the source of water.^84^ Moreover, significant changes were observed in *Acinetobacter* and *Staphylococcus* genera associated with antibiotic resistance.^84^

As another influencing factor, nonantibiotic drugs do not affect the gut microbiota alone, but interact with the gut microbiota to directly affect the individual's response to specific drugs, this phenomenon called pharmacomicrobiome.^85^ Common drugs that affect gut microbiota include proton‐pump inhibitors, which are the most associated with reduced diversity and taxonomic changes in the gut microbiota.^86^ *Enterococcus faecalis* and *Ruminococcus torques* can directly metabolize the drugs levodopa and entacapone for the treatment of PD.^87^

Of note, there are hundreds of intrinsic and environmental factors that influence the gut microbiota. There is an interaction between these influencing factors and gut microbes, and the effects of the interaction are likely to be complex. The relative importance and extent of influence of these factors may also vary depending on individual differences. The gut microbial community is a highly complex ecosystem, and more studies are still needed to fully understand its formation and regulation mechanisms.

## 3 GUT MICROBIOTA–BRAIN AXIS COMMUNICATION

### 3.1 Bidirectional communication between the gut and the brain

Gut microbiota–brain axis (GBA) communication refers to the connection network involving multiple biological systems, which realizes bidirectional communication between gut microbiota and brain. The gut–brain axis, mediated by several direct and indirect pathways, maintains the homeostasis of the host GI tract, CNS, and microbial system. It is also regarded as the pathophysiological mechanism behind certain neurological disorders. It is probable that multiple mechanisms and pathways work together in this process.^1^

Stress hormones, immune mediators, and central neurotransmitters can activate neuronal cells in the enteric nervous system (ENS) and vagus nerve (VN), thereby changing the gut environment and the structure of the gut microbiota.^8^, ^88^ Most studies in germ‐free (GF) mice and mouse models treated with broad‐spectrum antibiotics have revealed the effects of gut microbial signaling blockade on neurodevelopment and the induction of neurological disorders.^89^, ^90^, ^91^, ^92^ The lack of gut microbiota is even associated with increased blood–brain barrier (BBB) permeability and tight junction protein expression.^93^ In addition to the effects of gut microbial defects on the nervous system, wild mice that received microbiota from diseased mice also developed nervous system damage. For instance, wild‐type mice that were transplanted with gut microbiota from mice with AD exhibited colonic inflammation and memory function impairments.^94^ Rotenone‐induced PD mice can alleviate neuronal damage and inhibit neuroinflammation by transplanting gut microbiota from healthy mice.^95^ Certain psychiatric conditions such as major depression, anxiety, psychosis, schizophrenia, and bipolar disorder also exhibit dysbiosis in the gut microbiota, marked by a decrease in anti‐inflammatory bacteria and an increase in proinflammatory bacteria.^96^ However, the supplementation of prebiotics and probiotics has the potential to boost the levels of certain microbes on a temporary or permanent basis, characteristics the traits and functions of the microbiome, and engage with the host. Studies have demonstrated that probiotic intake can alleviate or prevent a range of ailments in both mouse models and humans, including neurological disorders.^97^, ^98^

These discoveries offer concrete evidence backing the concept of a two‐way communication between the gut and the brain. Nevertheless, it is crucial to note that the connection between the gut and the brain is intricate, and the field is continuously advancing, with ongoing research efforts.

### 3.2 Mechanisms of communication

**FIGURE 1:**
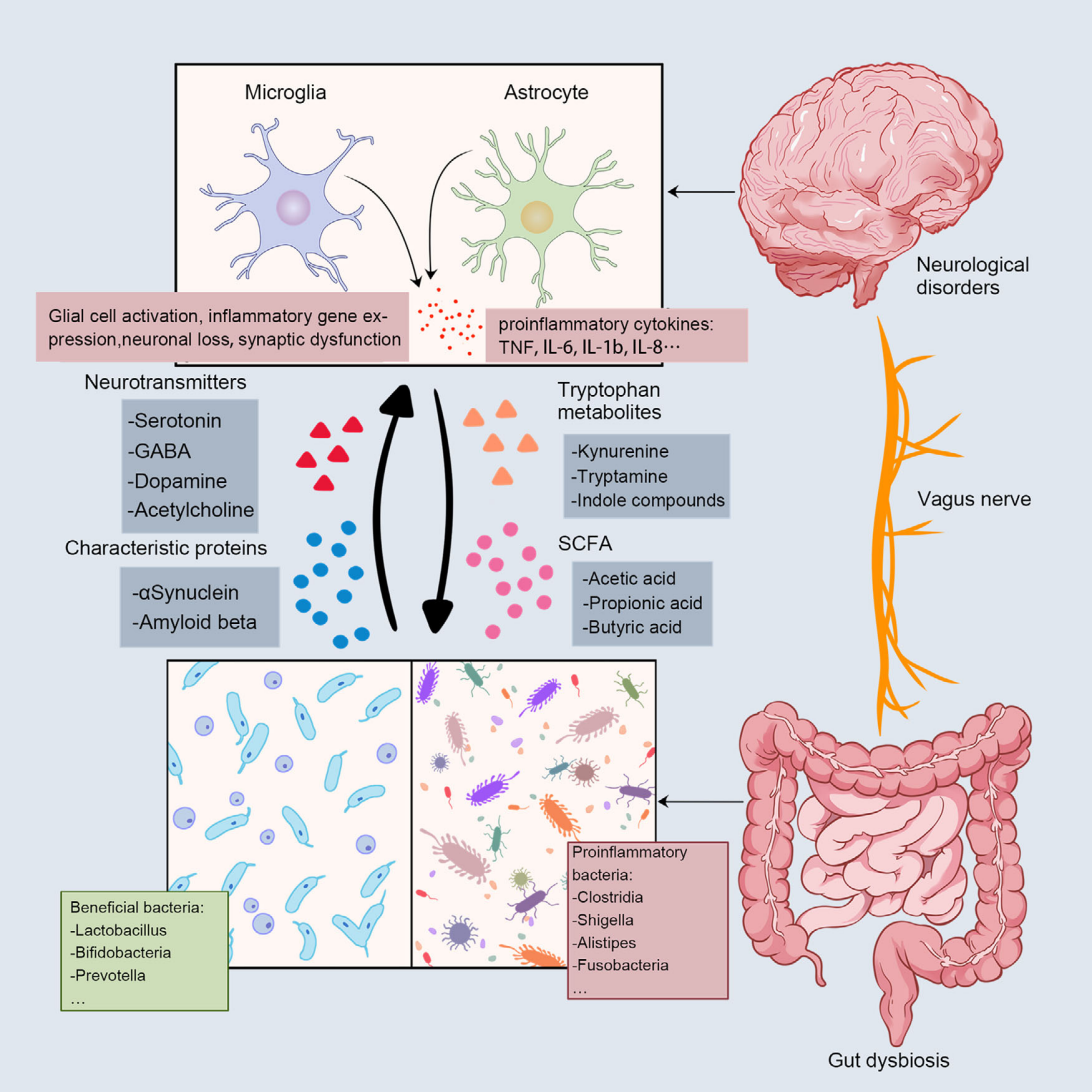
Bidirectional communication between the gut and the brain. The main communication pathways between microbes and the brain include neural pathways, immune pathways, and metabolic signals. Gut disorders send signals to the brain via the vagus nerve, and a decrease in beneficial bacteria and an increase in proinflammatory bacteria cause altered levels of microbial metabolites, including neurotransmitters, SCFA, and indole metabolites. The deposition of characteristic proteins in neurodegenerative diseases has also been associated with gut microbiota (Aβ in AD; αSyn in PD). These signals stimulate glial cells in the brain, the expression of proinflammatory genes, neuronal loss, synaptic dysfunction, and the rise of proinflammatory cytokines.

According to the current researches, the communication mechanism between the gut and brain is realized through a variety of pathways, mainly the nervous system, immune system, and metabolic pathways (Figure 1).

#### 3.2.1 Neural pathways

The nervous system is the main route of information transfer between the gut and the brain. The brain and gut communicate directly through the autonomic nervous system (ANS) and VN in the spinal cord. The ANS binds neuronal and neuroendocrine signals to induce CNS‐regulated gut changes through sympathetic, parasympathetic branches, and the hypothalamic–pituitary–adrenal (HPA) axis, affecting ENS, VN, neuroendocrine system, immune system, metabolic pathways, and gut microbiota.^1^, ^8^, ^99^ The VN is the longest parasympathetic nerve in the body and connects the brain stem to the ENS. The VN is able to sense gut information about the microbiome and transmit it to the CNS, and some strains can produce neurotransmitters or modulate neurotransmitters that act directly on VN terminals.^100^, ^101^ The cholinergic anti‐inflammatory pathway of VN fibers is able to suppress peripheral inflammation and reduce gut permeability, and it is also likely that the composition of the gut microbiota is therefore regulated.^100^ VN may also mediate the effects of gut bacteria on brain function and behavior. For example, the age‐related gut microbiota induces reduced neuronal activation and hippocampa‐dependent memory impairment in a VN‐dependent manner.^102^ Oral *Lactobacillus rhamnosus* treatment reduced anxiety‐ and depression‐related behaviors through VN in mice, but neither neurochemical nor behavioral effects were observed in VN‐cut mice.^98^

ENS, the largest branch of the ANS, is known as the “second brain” and consists of a tight network of neurons and glial cells. The main function of the ENS is to regulate the motility and secretory function of the GI tract. The development and function of the ENS are partly induced by the gut microbiota, and aryl hydrocarbon receptors (AHRs) can regulate gut peristalsis through the ENS.^8^ Intestinal epithelium, immune cells, and soluble neuroactive signals can transmit stimuli from the gut microbiota to the ENS through a variety of mechanisms.^103^ After the depletion of gut microbiota, the loss of gut neurons, the increase of intestinal permeability, and the decrease of gut glial cells occur. While when the gut microbiota spontaneously recovers, the altered GI physiology and enteric neuronal loss also recover.^91^ Moreover, glucocorticoid signaling in the ENS also appears to mediate the effects of chronic psychological stress on gut inflammation.^104^

#### 3.2.2 Immune signaling

The gut microbiota plays a central role in the establishment and homeostasis of the innate immune system.^105^ During the early stages of life, the establishment of gut microbiota allows intestinal dendritic cells to transfer microbial antigens from the gut to the thymus, triggering the proliferation of microbiota‐specific T cells.^106^ The composition of gut microbiota in newborns can impact the population and function of CD4 T cells through alterations in the gut microenvironment.^107^ In the human gut, plasma cells secrete IgA, and the mature affinity IgA system plays a central role in the interactions between secretory IgA and the gut microbiota in both healthy and inflamed adult gut.^108^ However, probiotic strains can enhance IgA secretion and function to protect barrier function.^109^ Microglia, the predominant monocyte–macrophages in the CNS, oversee brain development, uphold neuronal networks, and modulate immune responses in the CNS.^110^, ^111^ Evidence suggests that microglia are subject to regulation by the GBA. As individuals age, changes in the gut microbiota can modify the transcriptional profile of microglia, affecting genes related to interferon signaling, immune and inflammatory responses, as well as microglial migration.^112^ Furthermore, the gut microbiota has the ability to stimulate immune cells and engage in communication via cytokine and receptor interactions.^113^ Specific bacterial species can respond to immunological changes in different immune cells.^113^ Numerous lactobacillus species have the ability to promote the development of Tregs cells and decrease the production of inflammatory cytokines, contributing to immune regulation.^105^ *Bifidobacterium*, the most commonly mentioned probiotic, also has immunomodulatory ability. Not only its extracellular polysaccharides are related to the regulation of host immune response, but metabolites directly produced by *Bifidobacterium* are also an immune mediator.^114^ Being one of the largest immune organs in the human body, the intestinal mucosal immune system is responsible for safeguarding the intestine and preserving the integrity of the intestinal barrier. Research conducted in GF mice has revealed the pivotal role of the gut microbiota in the development of mucosal immunity. GF animals exhibited reduced numbers of intraepithelial lymphocytes, significantly fewer IgA‐secreting plasma cells in the lamina propria, and a lower abundance of Tregs compared with specific pathogen‐free animals.^115^ In addition, in vitro and in vivo experiments with probiotics have shown that probiotics can inhibit inflammatory pathways and inflammatory markers, and positively or negatively stimulate immune cells to regulate the immune system and inflammatory response.^116^

#### 3.2.3 Metabolic signaling

Microbial metabolism influences various mediators of the MGBA, such as neurotransmitters, SCFA, hormones, and immunomodulators. Neurotransmitters are essential chemical signals that neurons use to communicate with each other, playing a critical role in neural function and human behavior. Neurotransmitter abnormalities are closely linked to conditions like depression, anxiety, and mood disorders.^117^, ^118^ Neurotransmitters (dopamine, GABA, or serotonin) are produced not only by host cells but also by diverse gut microbiota.^119^, ^120^, ^121^ For example, altered serotonin (5‐HT) and associated changes in microbiota composition have been observed in irritable bowel syndrome, PD, and AD.^119^ Most gut bacteria (*Prevotella*, *Bacteroides*, *Lactobacillus*, *Bifidobacterium*, *Clostridium*) can indirectly affect the dopaminergic system and change the content of dopamine.^121^ GABA is expressed by *Bacteroides*, *Parabacteroides*, and *Escherichia* species and is an essential nutrient for *Ruminococcaceae KLE1738*.^120^ Furthermore, because GABA transporters are present in the BBB, gut‐produced GABA may even reach the brain.^122^ Mice that received gut microbiota transplants from individuals with schizophrenia displayed reduced brain glutamate levels and disturbances in glutamate–glutamine–GABA cycling, which were consistent with the pathological state of patients with schizophrenia.^123^

In addition to neurotransmitters, gut metabolites have effects on the brain and behavior. SCFA plays a key role in the MGBA crosstalk and the development and function of the CNS immune system. It is a classic gut microbiota metabolite and can regulate the BBB and neuroimmuno‐endocrine functions.^124^, ^125^, ^126^ The gut microbiota produces SCFA by fermentor carbohydrates and dietary fiber, the most common of which are acetic acid (C2), butyric acid (C4), and propionic acid (C3). However, the diversity of gut microbiota also determines the proportion of each SCFA produced.^125^, ^126^, ^127^, ^128^ SCFA has a direct inhibitory effect on the stimulated microglial inflammatory response and prevents or balances age‐related microglial dysfunction.^129^, ^130^ For example, butyrate attenuates the expression of inflammatory genes (IL‐1β, IL‐1rn, IL‐6, Nlrp3, Tlr4, and Tnf) in microglia of aged mice, ameliorates age‐related microbiota dysregulation, and limits colonic inflammation.^131^

Gut microbiota degrade dietary proteins and undergo tryptophan metabolism, which produces metabolites including serotonin, kynurenine (Kyn), tryptamine, and indole compounds.^132^ Indole and SCFAs, as ligands of AHR, can inhibit the activation of NF‐κB signaling pathway and the formation of NLRP3 inflammasome.^133^, ^134^ Indole derivative indole‐3 propionic acid can promote nerve regeneration and repair through neutrophil chemotaxis. Microbiota metabolites can directly affect the brain and behavior after being transported across the BBB, or indirectly affect the brain and behavior through neuroendocrine, immune, or vagal pathways.^88^

## 4 ROLE OF THE GUT MICROBIOTA–BRAIN AXIS IN NEUROLOGICAL DISORDERS

According to the data of some preclinical studies, the gut microbiota acts along the MGBA through the above three mechanisms, affecting the BBB permeability, synapse shaping, neurogenesis, and neuronal signaling, thereby affecting social ability, sensory, memory, learning, stress, and other behaviors and emotions.^9^, ^135^, ^136^ Several more discussed neurological disorders are discussed and summarized below.

### 4.1 Neurodevelopmental disorders

#### 4.1.1 Autism spectrum disorders

ASD, a neurodevelopmental disorder defined by impaired social communication and restrictive repetitive behaviors, is considered to be a collection of related disorders with different etiologies.^137^ ASD is a prevalent neurodevelopmental condition with a strong genetic component and also be associated with environmental risk factors (e.g., neonatal hypoxia, maternal obesity, pesticides, and air pollution).^138^ However, the exact etiology of ASD remains unclear and is still being explored. In recent years, it has been proposed that the occurrence and development of ASD is related to gut microbiota. Studies have shown that patients with ASD have characteristic dysbiosis of gut microbiota compared with healthy controls, with *Clostridium* species being one of the most frequently detected dysbiosis bacteria in ASD patients.^9^, ^139^ At the same time, *Proteobacteria*, as a microbial characteristic of gut microbiota imbalance, is also increased in ASD patients^139^ (Some studies concerning alterations in the composition of the human gut microbiota in ASD and other neurological diseases are shown in Table 2). Research with animals has demonstrated that gut microbiota taken from individuals with ASD can induce behavioral modifications in mice and even alter alternative splicing patterns of genes in the brains of recipient offspring mice (adult mice that inherit microbes from human donors).^140^ At the same time, the development of gut microbiota in the ASD group also showed a severe lag state in multiple aspects of diversity.^141^ There is evidence that the structural and functional maturation of microglia is associated with gut microbiota.^92^ ASD patients have been found to have increased microglia density and increased distance between microglia and neurons, potentially contributing to synaptic degeneration.^142^ Altered neural connections and synaptic dysfunction in ASD patients may contribute to impaired social communication and a core phenotype of repetitive behavior.^142^ This implies that changes in microglia and neuroinflammation in the CNS of individuals with ASD may be linked to disruptions in gut microbiota, highlighting the gut microbiota's role in influencing brain function.

**TABLE 2:** Alterations in human gut microbiota composition in neurological disorders.

Disease	Species	Increased taxa	Reduced taxa	References
ASD	Children with ASD	Phylum: Proteobacteria Genus: Clostridium, Enterococcus, Prevotella, Alistipes Species: Roseburia inulivorans, Roseburia sp.	Phylum: Firmicutes Genus: Faecalibacterium, Bifidobacterium Species: Roseburia intestinalis, Roseburia faecis,	^147^ This is a review.
ASD	Patients with ASD (*y* = 2–18)	Phylum: Proteobacteria, Fusobacteria, Verrucomicrobia Genes: Lactobacillus, Bacteroides, Desulfovibrio, Akkermansia	Genus: Bifidobacterium, Blautia, Dialister, Prevotella, Turicibacter, Veillonella, Akkermansia muciniphila	^139^ This is a systematic review.
ASD	Chinese children with ASD (*y* = 2–7, *n* = 48)	Genus: Bacteroides, Prevotella, Lachnospiracea_incertae_sedis, Megamonas Species: Bacteroides coprocola, Bacteroides vulgatus, Eubacterium eligens, Prevotella copri, Roseburia faecis	Phylum: Proteobacteria, Verrucomicrobia Genus: Clostridium, Escherichia/Shigella Species: Akkermansia muciniphila, Dialister invisus, Escherichia coli, Bacteroides fragilis, Haemophilus parainfluenzae, Flavonifractor plautii	^148^
ADHD	Chinese children and adolescents (*y* = 6–15, *n* = 98)	Family: Listeriaceae, Prevotellaceae, Veillonellaceae Species: Prevotella copri, Prevotella buccae, Bifidobacterium breve, Bifidobacterium bifidum	Species: Bacteroides ovatus, Bacteroides nordii, Bacteroides thetaiotaomicron, Bacteroides intestinalis, Bacteroides cellulosilyticus, Bacteroides fluxus	^149^
ADHD	Children with ADHD (*y* = 6–16, *n* = 41)	Genus: Desulfovibrio, Roseburia, Ruminococcaceae, Agathobacter, Anaerostipes, Lachnospiraceae UCG‐010	Genus: Bacteroides, Prevotella	^150^
ADHD	Children with ADHD (mean age: 8.4, *n* = 30)	Genus: Sutterella Fusobacterium, Alistipes, Species: Sutterella stercoricanis, Bacteroides uniformis, Bacteroides ovatus	Genus: Prevotella, Species: Bacteroides coprocola	^151^
AD	Chinese patients with AD (*y* = 50–85, AD, *n* = 33; aMCI, *n* = 32)	Phylum: Proteobacteria Class: Gammaproteobacteria Family: Veillonellaceae, Enterobacteriaceae	Phylum: Firmicutes, Bacteroidetes Family: Clostridiaceae, Lachnospiraceae, Ruminococcaceae, Blautia	^152^
AD	American patients with AD (mean age = 71.3, *n* = 25)	Phylum: Bacteroidetes, Proteobacteria Family: Gemellaceae, Bacteroidaceae, Rikenellaceae Genus: Blautia, Phascolarctobacterium, Gemella, Bacteroides, Alistipes, Bilophila	Phylum: Firmicutes, Actinobacteria Family: Bifidobacteriaceae, Ruminococcaceae, Turicibacteraceae, Clostridiaceae, Mogibacteriaceae Genus: Clostridiaceae SMB53, Dialister, Clostridium, Turicibacter, Erysipelotrichaceae cc115, Bifidobacterium, Adlercreutzia	^153^
AD	Patients with AD (AD: mean age = 66.3, *n* = 30; MCI: mean age = 65.4, *n* = 30)	Phylum: Firmicutes Genus: Lactobacillus, Akkermansia, Dorea, Bifidobacterium, Streptococcus, Acinetobacter, Blautia, Escherichia	Phylum: Bacteroidetes Genus: Parabacteroides, Alistipes, Bacteroides, Alloprevotella, Haemophilus, Paraprevotella, Sutterella, Prevotella, Barnesiella, Butyricimonas	^154^
PD	Patients with PD (*n* = 80)	Genus: Parabacteroides, Verrucomicrobia, Akkermansia, Butyricimonas, Enterococcus, Lactobacillus, Bilophila, Mucispirillum, Odoribacter, Veillonella	Genus: Prevotella	^155^
PD	Canadian patients with PD (*y* = 59–71, *n* = 197)	Family: Christensenellaceae, Desulfovibrionaceae Genus: Bifidobacterium, Collinsella, Bilophila, Akkermansia	Family: Lachnospiraceae, Genus: Roseburia, Faecalibacterium	^156^
PD	Italian patients with PD (mean age = 71.39, *n* = 64)	Phylum: Proteobacteria, Verrucomicrobia, Actinobacteria Family: Verrucomicrobiaceae, Bifidobacteriaceae, Streptococciaceae, Desulfohalobiaceae Genus: Akkermansia, Escherichia, Streptococcus, Clostridium, Bifidobacterium	Phylum: Bacteriodetes Family: Bacteroidaceae, Lachnospiraceae, Brevibacteriaceae, Sphingobacteriaceae Genus: Bacteroides, Blautia, Lachnospira, Roseburia, Coprococcus	^157^
Depression	Chinese patients with depression (*n* = 46)	Phylum: Bacteroidetes, Proteobacteria Family: Bacteroidaceae, Enterobacteriaceae, Porphyromonadaceae, Rikenellaceae Genus: Alistipes, Bacteroides, Parabacteroides, Phascolarctobacterium, Roseburia	Phylum: Firmicutes, Fusobacteria, Actinobacteria Family: Lachnospiraceae, Ruminococcaceae, Veillonellaceae Genus: Escherichia/Shigella, Oscillibacter, Dialister, Faecalibacterium, Prevotella, Ruminococcus	^158^
Depression	Chinese patients with depression (*n* = 165)	Phylum: Bacteroidetes Family: Bacteroidaceae, Veillonellaceae, Lachnospiraceae Genus: Bacteroidetes	Family: Enterobacteriaceae, Pseudomonadaceae, Ruminococcaceae, Christensenellaceae	^159^
Anxiety	Chinese patients with anxiety (*n* = 36)	Order: Betaproteobacteriales, Enterobacteriales Family: Bacteroidaceae, Enterobacteriaceae Genus: Bacteroides, Escherichia/Shigella,	Phylum: Firmicutes Family: Prevotellaceae, Genus: Dialister, Subdoligranulum, Megamonas, Acinetobacter	^160^
Anxiety	Chinese patients with anxiety (*n* = 40)	Phylum: Fusobacteria, Bacteroidetes Genus: Fusobacterium, Ruminococcus gnavus, Bacteroides	Phylum: Firmicutes Genus: Lachnospira, Butyricicoccus, Sutterella, Eubacterium rectale, Faecalibacterium	^161^

Abbreviation: aMCI, amnestic mild cognitive impairment.

Significant variations in the functional components of gut microbiota were observed between individuals with ASD and those without. ASD patients exhibited notable alterations in tryptophan and serotonin metabolism, as well as reduced levels of taurine and 5‐aminolevulinic acid.^134^, ^140^, ^143^ Moreover, approximately 40% of children diagnosed with ASD experience GI issues such as abdominal pain, diarrhea, bloating, and gastroesophageal reflux, whereas prevalence of GI symptoms in children with ASD may even reach 70%.^88^, ^122^ This implies that children with ASD are more likely to have “gut leakage” and that the CNS is highly exposed to proinflammatory cytokines due to this higher permeability.^9^ For example, tumor necrosis factor (TNF), proinflammatory cytokines (IFN, IL‐1b, IL‐6, IL‐8, IL‐p4) were elevated in the brains of children with ASD, and even higher levels of IgA were present.^144^

Collectively, these results demonstrate the influence of gut microbiota in ASD and also suggest the potential therapeutic application of gut microbiome in ASD. In fact, there are no effective medications reported to treat the core symptoms of ASD, and more individualized treatments are available for different individuals, including behavioral therapy, physical therapy, drug therapy, and educational support. Currently, certain research studies have utilized probiotics and prebiotics for intervention in ASD, assessing their impact on behavior scores and the gut microbiome. For instance, in a study involving galactose oligosaccharides and an exclusion diet, antisocial behavior scores notably decreased in individuals with ASD, accompanied by alterations in *Bifidobacterium* and butyrate‐producing bacteria.^145^ *Lactobacillus reuteri* treatment ameliorated ASD‐like social deficits in mice in a vagus‐dependent manner.^146^ However, patients with ASD have strong clinical heterogeneity and etiologic complexity. Although microbiota modulation is a promising option for the treatment of ASD‐related behaviors and GI symptoms, the development of broad and effective gut microbiota agents is almost impossible to achieve. Subsequent research efforts should concentrate on improving the precision of biomarker tests and the development of specific psychoprobiotic drugs targeting the gut microbiota of ASD patients, leading to the establishment of personalized treatment plans.^135^

#### 4.1.2 Attention‐deficit/hyperactivity disorder

ADHD is a childhood‐onset neurodevelopmental disorder characterized by inappropriate development and inattention, motor hyperactivity, and impaired impulsivity.^162^ Moreover, the biological mechanism of ADHD neurodevelopment is still unclear, and there are still no clear diagnostic biomarkers. In contrast to research on ASD, there is a scarcity of well‐defined and consistent studies exploring the relationship between ADHD and gut microbiota. Both human and animal studies have indicated that the differences in α diversity and β diversity analysis findings between individuals with ADHD and those without the condition are not statistically significant.^149^, ^150^, ^163^ Studies have also shown that there are differences in the composition of gut microbiota between ADHD patients and healthy people.^150^ Increased relative abundance of *Acteroides nordii*, *Bacteroides cellulosilyticus*, and *Bacteroides intestinalis* was found to be associated with decreased symptoms of hyperactivity/impulsivity deficits and attention deficits.^149^ *Bacteroides thetaiotaomicron* and *Bacteroides ovatus* were only negatively correlated with attention deficits.^149^ Anxiety was higher in mice colonized with the ADHD patient's microbiome, but the mice had no memory deficits or impulsive behavior.^163^ However, in several other studies, no differences were found between ADHD patients and controls.^164^ Patients with ADHD exhibit elevated levels of proinflammatory markers, leading to investigations on the potential impact of gut microbiota on proinflammatory cytokine levels in this population. Research indicates a notable decrease in plasma TNF‐α levels in individuals with ADHD compared with healthy controls, with TNF‐α levels showing a negative association with ADHD symptoms and gut microbiome diversity.^150^ It has been shown that the level of TNF‐α is negatively correlated with the correlation of attention deficit and hyperactive/impulsive symptoms.^150^ However, discrepancies exist regarding the relationship between TNF‐α levels and ADHD symptoms. Some studies suggest a lack of significant correlation between TNF‐α levels and ADHD diagnosis, whereas others indicate a positive correlation between TNF‐α levels and ADHD scores.^150^, ^165^ The constraints and variability observed in these pertinent studies could be attributed to factors such as the age of the study participants, racial diversity, geographical variations, lifestyle factors, and disparities in research methodologies.^166^ Similarly, compared with ASD, there are fewer studies on ADHD trying to improve the condition by probiotics or prebiotics. In a randomized controlled trial, researchers conducted a 9‐week intervention with a pre + probiotic called synbiotic 2000 (include three lactic acid bacteria and four dietary fibers) in children and adults with ADHD.^167^ In children with ADHD, synbiotics, as compared with placebo, reduced plasma markers of proinflammatory activity by increasing SCFA levels, which the investigators defined as a suggestive difference.^167^ In adults, the synbiotic 2000 treatment effect was not statistically significant or suggestive for the entire group.^167^ Since some participants were still using ADHD medications, the alterations in plasma immune activity markers in ADHD patients could be attributed to the influence of these medications or the age and gender of the patients. Nevertheless, no distinction in treatment effectiveness was observed between the synbiotic 2000 intervention and the placebo, and both were effective in alleviating ADHD symptoms.^168^ Specifically, synbiotic 2000 was able to reduce symptoms of autism in children without drug treatment and improved emotion regulation in adults with ADHD.^168^ Specifically, synbiotic 2000 was able to reduce symptoms of autism in children without drug treatment and improved emotion regulation in adults with ADHD.^168^

The cause and diagnosis of ADHD are intricate, lacking a definitive diagnostic indicator, with concurrent symptoms of depression or anxiety. The investigation into the gut microbiome as a novel avenue for diagnosing and treating ADHD remains limited, necessitating numerous additional studies to delve into the role of gut microbes in ADHD.

### 4.2 Neurodegenerative disorders

#### 4.2.1 Alzheimer's disease

AD is a neurodegenerative disease manifested by impaired cognition and memory. It is the most common cause of dementia, with one of the main features being the accumulation of extracellular plaques containing β‐amyloid protein (Aβ) and intracellular tau‐containing neurofibrillary tangles (NFTs).^169^ The involvement of gut microbiota in AD research has been a topic of discussion for a while, with a substantial body of research evidence emerging. Animal studies have shown that FMTs from AD mice can induce memory impairment and neuroinflammation in wild‐type mice.^87^ FMT from aged APP/PS1 mice lead to elevated levels of gut β‐secretase (BACE1) and Aβ42, which induce neuroinflammation and early AD pathology in wild‐type mice.^170^ Even individuals of young healthy mice who received FMT from AD patients developed AD behavioral phenotypes.^171^ However, long‐term administration of FMT from healthy wild‐type mice can alleviate Aβ deposition, tau pathology, and memory impairment in AD mice.^172^ Of note, as the most prevalent genetic risk gene for AD, apolipoprotein E (ApoE)‐mediated neuroinflammation is involved in tau‐mediated neurodegeneration, and ApoE genotype is associated with differences in the abundance of several gut bacterial taxa, particularly the butyrate‐producing gut microbiota, which may drive differences in the metabolite levels of the gut microbiota.^173^, ^174^ Emerging evidence using a mouse model of taupathy expressing the human ApoE isoform and manipulation of the gut microbiota with being raised in GF conditions or short‐term antibiotic (ABX) treatment suggests that the gut microbiota regulates neuroinflammation in an ApoE isoform‐dependent manner.^175^

As individuals age, alterations in the balance between the host and gut microbiota occur, influencing the rate of decline in both physical function and cognition. In other words, the decline in gut microbiota diversity or the presence of gut dysbiosis due to aging may contribute to the progression of AD. General changes associated with aging are characterized by the loss of dominant commensal bacteria; these taxa may be dominated by second symbionts and pathogenic bacteria.^18^, ^176^, ^177^ The composition and relative abundance of gut bacteria from phylum to genus are different in AD patients compared with healthy people. The structural changes of gut microbiota in AD patients are mainly reflected in the increase of pathogenic bacteria from some *Proteobacteria* and the decrease of *Firmicutes* and *Actinobacteria* in the gut microbiota of AD.^8^, ^152^, ^153^, ^178^, ^179^ Cognitive decline was associated with the loss of diversity in the gut microbiota, with a reduction in beneficial bacteria diversity (*Lactobacillus* and *Bifidobacteria*) and an increase in many proinflammatory bacteria (such as *Propionibacteria, Fusobacteria, Shigella*, and *Clostridia*).^180^ Among these, the *Clostridiales subpopulation* associated with cognitive decline significantly, and much higher than the loss of gut microbial diversity.^181^ SCFAs derived from the metabolism of the gut microbiota play a significant role in the onset and progression of AD. For example, acetate, a SCFA produced by gut bacteria, promotes microglial maturation, regulates the homeostatic metabolic state, and modifies the pathology of AD by influencing microglial innate immune mechanisms.^182^ Propionate can effectively improve cognition and memory in AD mice by reducing the activation of proinflammatory cytokines and increasing the expression of synapse‐associated proteins.^183^

The inflammatory reaction within the CNS, primarily orchestrated by microglia and astrocytes, is believed to play a crucial role in AD development, similar in importance to Aβ accumulation and NFTs. Neuroinflammation can arise as a result of CNS injury, infection, toxicity, and autoimmune responses.^184^, ^185^ AD‐related gut microbiota can upregulate the metabolism of proinflammatory polyunsaturated fatty acids, enhance the activation of microglia and aggravate neuroinflammation by activating the C/EBPβ/AEP pathway.^186^ During aging, gut microbiota alter the microglial transcriptional family profile and increase levels of a major advanced glycation end product (AGE), N6‐carboxymethyllysine (CML), by disrupting gut permeability. This age‐related accumulation of CML directly induces microglial metabolic dysfunction that affects brain function.^112^ Similar to microglia, astrocytes release cytokines, interleukins, nitric oxide, and other potentially cytotoxic molecules upon activation, exacerbating the neuroinflammatory response.^184^ Furthermore, gut microbiota metabolites can also drive astrocyte phenotypes. Increased SCFA can inhibit the activation and proinflammatory phenotype of astrocytes.^187^ And indole has the potential to suppress the synthesis of proinflammatory cytokines in astrocytes and lessen central neuroinflammation.^133^

Current evidences suggest that inflammatory enteritis is associated with a high risk of AD.^188^ Intestinal permeability defects are the basis of chronic low‐grade inflammation; early dysbiosis may alter the properties of the intestinal barrier; and translocation of intestinal contents may directly or indirectly affect CNS function through one or more pathways.^189^ Furthermore, circulating bacterial product levels increased with age.^190^ In addition, GI tract infection can lead to bacterial translocation to the intestinal mucosa, produce inflammation, further aggravate intestinal barrier dysfunction, and lead to a vicious cycle.^191^ In the process of GBA dysfunction and neuroinflammation caused by gut inflammation, the phenomenon of “gut leakage” has become a non‐negligible hypothesis supporting the pathophysiology of cognitive impairment, neurodegenerative diseases and many chronic diseases.

In summary, there are many potential mechanisms of gut microbiota in the pathological development of AD. When considering the intervention and adjuvant treatment of AD by gut microbiota, the complex network of the MGBA needs to be further explored to fully characterize the effect of gut microbiota on AD.

#### 4.2.2 Parkinson's disease

PD is also a common neurodegenerative disorder, mainly characterized by the early and significant death of dopaminergic neurons in the substantia nigra pars compactness, with typical symptoms including bradykinesia, muscle stiffness, resting tremor, and postural and gait disorders.^192^ At first, Braak's hypothesis suggested that PD begins in the gut.^193^ Subsequent studies have shown that the gut microbiota is associated with PD phenotypes, such as onset time, duration, disease stage, and clinical symptoms (motor and nonmotor).^155^, ^194^ It has been reported that GI disorders and gut dysbiosis occur before the onset of motor symptoms in PD.^195^ Aggregation of α‐Synuclein (αSyn) is a characteristic protein in PD, which usually leads to motor dysfunction.^196^ It has been found that αSyn is ubiquitously deposited in the GI tract and can be transmitted from the gut to the brain via the VN.^197^, ^198^ Although the presence of gut microbiota is necessary for motor impairments, microglial activation, and αSyn pathology, SCFA alone are adequate to stimulate αSyn‐related neuroinflammation.^196^ FMT in PD mice will also result in impaired motor function.^196^ Dysbiosis of gut microbiota and microbial metabolites in Parkinson's patients, including loss of bacteria associated with anti‐inflammatory properties (*Prevotella*, *Blautia*, *Coprococcus*, and *Roseburia*) and a shift in gut microbial balance to a more inflammatory phenotype.^199^, ^200^, ^201^ This may reduce the production of SCFAs and produce more endotoxin and neurotoxins. *Prevotella* is a highly specific precursor marker of PD, and constipation is lowest in individuals with enterotype enriched for *Prevotella*.^202^ In contrast, the decrease in *Prevotella* may be related to decreased mucin synthesis, which is associated with increased intestinal permeability.^203^ The relative abundance of *Verrucomicrobes* and *Bacteroidetes* is increased or correlated with motor symptom severity in PD patients and with elevated plasma TNFα and IFNγ concentrations in PD patients, which are also associated with inflammation in PD.^155^ In addition, a positive relationship has been found between the abundance of *Enterobacteriaceae* and the severity of postural instability and gait difficulty, suggesting that one or more changes in specific bacteria may be associated with the symptoms or pathology of PD.^204^ Decreased levels of SCFAs in feces, elevated SCFAs in plasma, and higher plasma concentrations of aromatic amino acid metabolites in PD patients with constipation are linked to particular changes in gut microbiota and the clinical severity of PD.^205^, ^206^

GI dysfunction, a characteristic feature of PD, is associated with neurodegeneration in ENS, mainly constipation (87%).^207^, ^208^ GI dysfunction is the earliest prodromal symptom, which predates the onset of movement disorders in PD by many year.^197^ There is a complex relationship between microbiota composition and gut function. The rise of detrimental proteolytic microbial metabolites in PD patients microbiota is strongly associated with constipation; however, there exists an inverse relationship between butyrate‐producing bacteria and constipation.^156^ However, the underlying pathogenesis of coordinated defecation disorders may involve dopaminergic dysfunction.^197^ Loss of dopaminergic neurons and reduced striatal dopamine levels underlie motor deficits in PD.^209^ Levodopa has been the first‐line drug for the treatment of PD. Gut bacteria have the ability to produce levodopa, and through circulation into the brain, it is converted to dopamine.^210^ *E. faecalis* or *Enterococcus faecium* transplanted into mice with PD significantly increased brain dopamine and improved PD performance.^210^

The role of GI tract and gut microbiota in the etiology of PD suggests the potential of gut regulatory intervention for PD treatment. This suggests that gut microbiota may be a new diagnostic tool and therapeutic target for PD. In a randomized, double‐blind, placebo‐controlled trial, probiotics in patients with PD for 12 weeks improved biomarkers of inflammation, oxidative stress, and insulin metabolism and lowered values on the Movement Disorders Society Unified Parkinson's Disease Rating Scale (MDS‐UPDRS) in the intervention group.^211^ *Lactobacillus plantarum CCFM405* ameliorated motor deficits and constipation, reduced dopaminergic neuron death, and reduced gut inflammation and neuroinflammation in rotenone‐induced PD mice.^212^ Furthermore, the MD, known for its anti‐inflammatory and antioxidant properties, has demonstrated efficacy in shielding patients from the early symptoms of PD, potentially slowing down the progression of the condition.^213^ However, finding a cure for PD remains a significant challenge. Present efforts in research have predominantly centered on the microbiome, overlooking the potential interplay between the ANS and the CNS that could underpin all etiological mechanisms.^207^ Subsequent research will be crucial in delving deeper into the connection between PD and gut microbiota, tailoring treatment to individual patients, and enhancing the design of clinical trials.

### 4.3 Mood disorders

#### 4.3.1 Depression

Depression, recognized as a prevalent mental health issue and affective disorder, has evolved into a significant public health concern with broad‐reaching effects across various age groups and socioeconomic statuses. Risk factors for depression primarily revolve around cognitive vulnerability, stressors, parental depression, interpersonal dysfunction, and prevalent among the female population.^214^ There are two main classification diagnostic systems for depression: the Diagnostic and Statistical Manual of Mental Disorders (DSM) and the International Classification of Diseases.^215^ Nonetheless, depression can manifest in a variety of forms with varying arrays of symptoms, may cooccur with other mental disorders, and the presentation of depressive symptoms seems to change over time for each patient.^216^, ^217^ But so far, there are no reliable biomarkers for major depressive disorder (MDD) and other types of depression.

Nonetheless, depression can manifest in a variety of forms with varying arrays of symptoms, may cooccur with other mental disorders, and the presentation of depressive symptoms seems to evolve over time for each patient. *Enterobacteriaceae* and *Alistipes* are highly prevalent in patients with depression, whereas *Faecalibacterium* is negatively correlated with depressive symptoms.^158^ Patients with MDD have an increased *Bacteroidetes/Firmicutes* ratio, characterized by an enrichment of *Bacteroidetes* and depletion of *Blautia*, *Faecalibacterium*, and Coprococcus.^218^ *Faecalibacterium faecalis* and *Coprococcus faecalis* are butyrate producing bacteria that enhance epithelial defense barriers and reduce gut inflammation.^118^ In addition, *Bacteroides* enterotype has potential dysbiosis properties, and higher *Bacteroides* enterotype is associated with depression.^118^

*Turicibacterales*, *Turicibacteraceae*, and *Turicibacter* are reduced in depression, and these bacteria may regulate secondary bile acid levels, which are negatively correlated with the severity of depressive symptoms.^218^ Circulating levels of proline were associated with microbial traits, including *Parabacteroides* and *Prevotella* genera, and were positively correlated with depression scores.^219^ The results of two‐sample two‐way MR analysis study showed no significant association between *Gammaproteobacteria* and MDD, but animal models found that increased levels of *Gammaproteobacteria* were associated with increased risk of MDD, and fluoxetine treatment was effective. This implies a strong correlation between the gut microbiota and anxiety‐ and depression‐like behaviors.^117^, ^220^ It has been reported that specific classes of antidepressants can exert different mechanisms of action on the gut microbiota.^218^, ^221^ Notably, the prevalence of anxiety and depression soared after the COVID‐19 pandemic, with expansion of *Proteobacteria* and depletion of *Synergistetes* phyla observed in individuals with depressive symptoms.^222^ However, the relative abundance of *Fusicatenibacter saccharivorans* was significantly reduced in individuals with comorbid symptoms of PTSD + depression + anxiety.^222^ Chronic, low‐grade inflammation is a hallmark of depression. The upregulation of NLRP3 inflammasome is related to the occurrence of depressive symptoms, and inhibiting NLRP3 inflammasome is an effective method for the treatment of depression.^223^, ^224^ Most animal studies have shown that the activation and inhibition of NLRP3 inflammasome by gut microbiota can regulate depression‐like behavior and affect hippocampal neuroinflammation and glial cell function in rodents.^225^, ^226^, ^227^

Gut microbiota is one of the influencing factors of depression, suggesting that microbial‐targeted therapy also has application prospects in alleviating depression. FMT of healthy adolescent volunteers to adolescent depressed mice significantly improved the depressive behavior of the mice, in which *Roseburia* played a key role.^228^ Effective colonization of *Roseburi*a in the mouse colon resulted in significant increases in 5‐HT levels and reciprocal reductions in the levels of Kyn toxic metabolites quinolonic acid and 3‐hydroxykynurenine in the mouse brain and colon.^228^ Some probiotics that can produce and deliver neuroactive substances, called psychoprobiotics, are beneficial to relieve depressive symptoms.^229^ *Lactobacillus* and *Bifidobacterium* are the most common probiotics in reducing depression. However, gut microbiota is affected by confounding factors, and negative results have also been reported.^98^, ^230^, ^231^, ^232^, ^233^, ^234^ In sum, these studies highlight the role of the gut microbiome in depression and suggest that the gut microbiome also holds promise as a therapeutic tool.

#### 4.3.2 Anxiety

Anxiety disorder is a common mental health problem characterized by excessive fear, anxiety, or avoidance of the environment. According to the DSM, fifth edition, there are different classification diagnostic criteria.^235^ In addition, anxiety is often a comorbidity of ASD, ADHD, and depression.^236^, ^237^, ^238^ Patients with depression often experience symptoms of anxiety, with the regulation of GBA function being linked to stress‐induced neurological symptoms, particularly anxiety.^12^ The high prevalence of comorbidity between anxiety and GI disorders further demonstrates the importance of the GBA in pathophysiology.^12^ According to the HPA axis imbalance theory, hormone imbalance is closely related to anxiety and stress disorders.^239^ Chronic stress‐induced anxiety is also regulated by the GBA.^240^ In a systematic review of gut microbiota in anxiety disorders, despite inconsistent results on microbial α and β diversity, specific bacterial taxa were associated with anxiety and depression, such as lower *Bacteroidetes*, *Prevotellaceae*, *Faecalibacterium*, and *Sutterella*, whereas *Enterobacterales*, *Enterobacteriaceae*, *Escherichia/Shigella*, and *Lactobacillus* were higher.^241^ A study of social anxiety disorder (SAD) showed that SAD FMT specifically increased sensitivity to social fear in recipient mice.^242^ Compared with healthy controls, SAD exhibited elevated relative abundance of *Anaeromassillibacillus* and *Gordonibacter* genera, whereas *Parasuterella* was enriched in SAD.^243^ Alterations in the microbiota caused during the critical period of life development have a significant and enduring influence on the diversity and structure of gut microbiota. Adolescent mice demonstrate heightened anxiety, specific alterations in circulating immune cells, and changes in adolescent neurophysiology.^244^ However, systematic reviews of the effects of gut microbiota depletion on sociability versus anxiety‐like behaviors in rodents have not yielded consistent results.^245^ This may be related to mixed or single setting antibiotics (ABX) treatment choice, differences in mixed or single setting ABX treatment choice, differences in study design and behavioral test parameters, rodent strains, study design and behavioral test parameters, and most importantly, the dynamics and complexity of the gut microbial spectrum.^245^ COVID‐19 infection and vaccination affected the overall microbial composition, with lower alpha diversity in individuals with trait anxiety symptoms.^222^ However, in the individuals with comorbid depression and anxiety after the COVID‐19 epidemic mentioned above, the decrease of *F. saccharivorans* may be associated with a decrease in the levels of the antidepressant acetylcarnitine, which may promote and/or exacerbate anxiety symptoms.^222^

## 5 MODULATION OF THE GUT MICROBIOTA FOR THERAPEUTIC INTERVENTION

**FIGURE 2:**
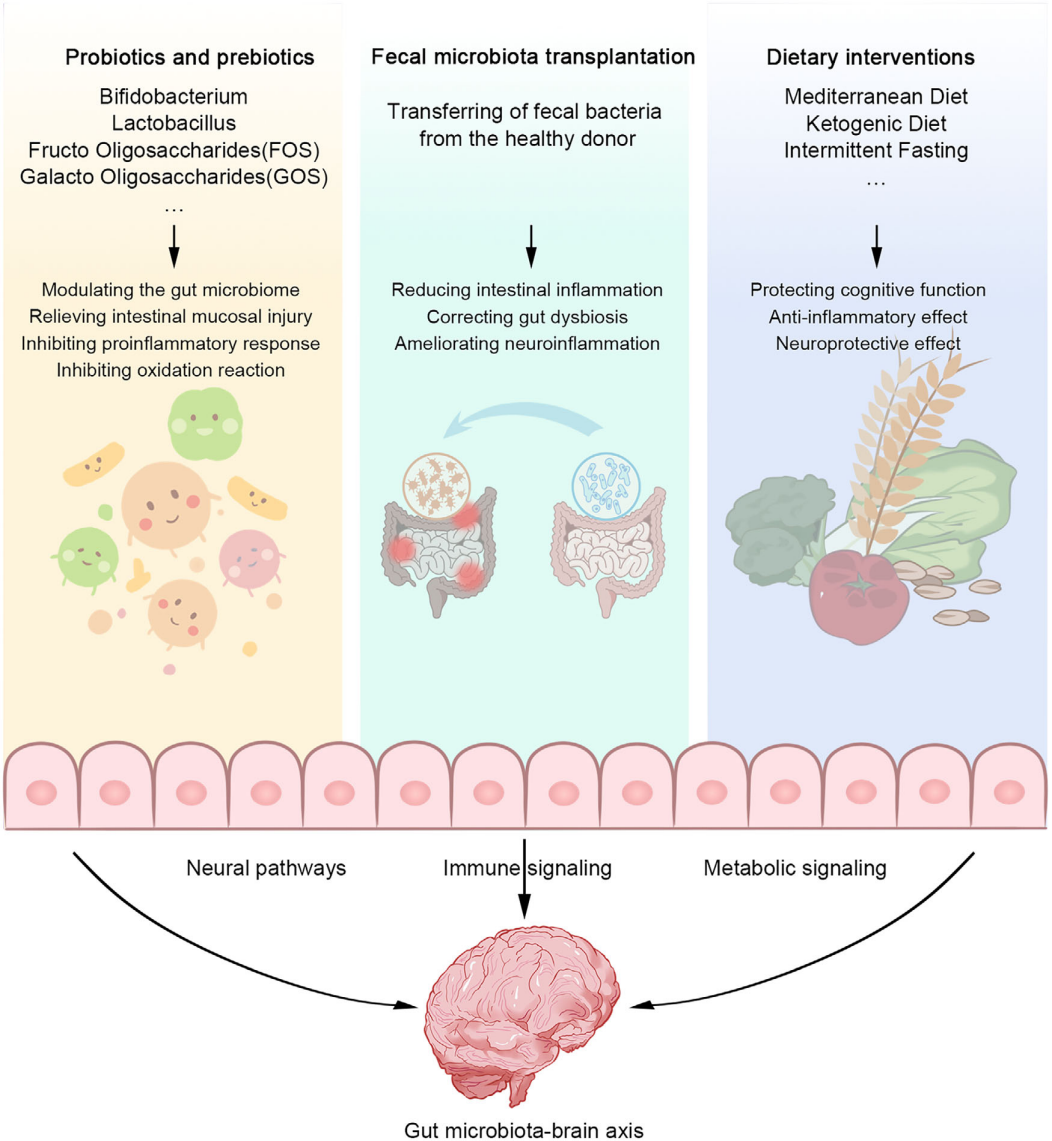
Therapeutic interventions to modulate the gut microbiota. Supplementation with probiotics and prebiotics (e.g., Bifidobacterium, Lactobacillus, FOS, GOS) regulates gut microbiota, reduces intestinal mucosal injury, inhibits proinflammatory response, and inhibits oxidative reaction. FMT reduces intestinal inflammation, corrects gut disorder, and ameliorates neuroinflammation. Dietary interventions (Mediterranean diet, ketogenic diet, and intermittent fasting) can protective cognitive function, anti‐inflammatory effects, and neuroprotective effects. These therapeutic interventions act on the gut–brain axis through neural, immune, and metabolic signaling pathways to improve gut health and, in turn, neurological disorders progression.

At present, the common therapeutic interventions by regulating gut microbiota are mainly probiotic and prebiotic supplementation, FMT, and dietary modification (Figure 2). Most studies have attempted to demonstrate that these therapeutic interventions can treat or delay neurological disorders by modulating gut microbiota (Table 3). In this section, we describe the specific content of these therapeutic interventions.

**TABLE 3:** Modulation of the gut microbiota for therapeutic intervention.

Diseases	Subjects	Operation	Results	References
**Probiotics**				
AD	AD patients	*Lactobacillus rhamnosus HA‐114* or *Bifidobacterium longum R0175*	Probiotic supplementation improved cognitive status in AD patients.	^246^
	AD patients	Multistrain probiotic supplements	Delayed the development of cognitive dysfunction.	^247^
	APP/PS1 mice	*Bifidobacterium breve HNXY26M4*	Reduce cognitive defects in mice, inhibit nerve inflammation and synaptic dysfunction, and regulating intestinal steady state.	^248^
	d‐galactose/AlCl_3_‐induced AD rats	*Akkermansia muciniphila*	Alleviated cognitive impairment, reduced the deposition of Aβ1‐42, and increased the abundance of SCFA‐producing bacteria.	^249^
	APP/PS1 mice	*Saccharomyces boulardii*	*Saccharomyces boulardii* treatment improved dysbiosis, alleviated the neuroinflammation as well as synaptic injury, and ultimately improved cognitive impairment.	^250^
PD	PD patients	*Lacticaseibacillus paracasei strain Shirota* (LcS)	LcS intervention significantly alleviated patients' constipation‐related symptoms and nonmotor symptoms.	^251^
	Rotenone‐induced PD mice	*Lactobacillus plantarum CCFM405*	*Lactobacillus plantarum CCFM405* ameliorated motor deficits and constipation, decreased dopaminergic neuronal death, reduced intestinal inflammation and neuroinflammation.	^212^
	MPTP‐induced PD mice	*Lactobacillus plantarum DP189*	*Lactobacillus plantarum DP189* could delay the neurodegeneration caused by the accumulation of α‐SYN via suppressing oxidative stress, repressing proinflammatory response, and modulating gut microbiota.	^252^
	MPTP‐induced PD mice	*Pediococcus pentosaceus* (PP)	PP treatment improved the gut microbial dysbiosis and increased the level of GABA.	^253^
	6‐Hydroxydopamine (6‐OHDA)‐induced PD rats	*Lactobacillus salivarius subsp. salicinius AP‐32* (AP‐32), residual medium (RM), and combination of AP‐32 and RM (A‐RM)	AP‐32, RM, and A‐RM supplementation induced neuroprotective effects on dopaminergic neurons along with improved motor functions.	^254^
ASD	ASD patients	SB‐121, a combination of *Lactobacillus reuteri*, Sephadex® (dextran microparticles)	Vineland‐3 measures showed significant improvement in adaptive behavior.	^255^
	MIA mice	*Lactiplantibacillus plantarum N‐1* (LPN‐1)	Reduced autism‐like behaviors, enhancd the relative abundance of the pivotal microorganisms of *Allobaculum* and *Oscillospira*, while reducing those harmful ones like *Sutterella* at the genus level.	^256^
	Rodent model of autism	bee pollen and probiotic‐treated	The synergistic treatment of bee pollen and probiotics showed neuroprotective and antioxidant effects.	^257^
	ASD mice	*Lactiplantibacillus plantarum ST‐III*‐fermented milk	*Lactiplantibacillus plantarum ST‐III* can help improve social behavior in a male mouse model of ASD and contribute to more balanced intestinal homeostasis.	^258^
ADHD	ADHD patients	*Bifidobacterium bifidum* (Bf‐688)	Symptoms of inattention and hyperactivity improved, and the composition of the gut microbiota was markedly altered.	^259^
	Spontaneously hypertensive rats (SHRs)	*Bifidobacterium animalis subsp. lactis A6* (BAA6)	BAA6 improved memory function by ameliorating hippocampal damage, abnormal neurotransmitter release and cerebral inflammation.	^260^
Anxiety	Anxious patients	*Lactobacillus helveticus R0052* and *Bifidobacterium longum R0175*	Increase total SCFAs and the secretion of anti‐inflammatory, as well as decreased proinflammatory cytokines.	^261^
Depression	MDD patients	Multispecies probiotics	Multispecies probiotics affects the stool metabolomic profile.	^262^
	MDD patients	*Bifidobacterium breve CCFM1025*	*Bifidobacterium breve CCFM1025* attenuated depression and gastrointestinal disorders.	^263^
	Chronic unpredictable mild stress (CUMS) model mice	*Lactobacillus reuteri strain 8008*	*Lactobacillus reuteri* attenuated depressive‐like behavior, improved blood lipids and insulin resistance, reduced inflammation in liver and adipose tissues, improved intestinal tight junctions.	^264^
	Social defeat stress (SDS) mice	*Lactobacillus paragasseri OLL2809*	OLL2809 ameliorated depression‐like behaviors, induced neurite outgrowth in the hippocampal dentate gyrus, and the abundance of *Akkermansia muciniphila*, *Bifidobacterium*, and *Lactobacillus* were increased by OLL2809 treatment.	^265^
**Prebiotics**				
AD	5×FAD mice	Mannan oligosaccharide (MOS)	MOS attenuated the cognitive deficits, reshaped microbiome and enhanced SCFAs formation. MOS balanced the brain redox status and suppressed the neuroinflammatory responses.	^266^
	(APP/PS1) transgenic Tg mice	Fructo‐oligosaccharides (FOS)	FOS treatment ameliorated cognitive deficits and pathological changes in the Tg mice.	^267^
PD	PD patients	Resistant starch (RS)	RS intervention increased fecal butyrate concentrations significantly, dropped fecal calprotectin concentrations, and reduce in nonmotor symptom load.	^268^
ASD	BTBR mice	Prebiotic (PRE; 10% oligofructose‐enriched inulin, Orafti®Synergy1, Beneo, Mannheim, Germany)	Probiotic and symbiotic treatments improved sociability and repetitive behavior.	^269^
Depression and anxiety	Chronic restraint stress (CRS) mice	Fructo‐oligosaccharides (FOS) and Galacto‐oligosaccharides (GOS)	The gut microbiota, gut and blood–brain barrier, and inflammatory response may mediate the protective effects of prebiotics on anxiety‐like behaviors.	^270^
**FMT**				
AD	5×FAD mice	Oral gavage in Old and Young 5×FAD recipient mice from healthy B6SJL wild‐type donor mice using fecal matter	“Plaque‐busting” and behavior‐modifying effects in treated 5×FAD.	^271^
	Sprague–Dawley rats	FMT from control subjects and Alzheimer's patients	Alzheimer's symptoms can be transferred to a healthy young organism via the gut microbiota.	^171^
	ADLP^APT^ mice	Transplantation of the fecal microbiota from WT mice into ADL^PAPT^ mice	Transplantation ameliorated the formation of amyloid β plaques and neurofibrillary tangles, glial reactivity and cognitive impairment.	^172^
	AD patients	Transplanted fecal microbiota were obtained from donors without GI or other health problems	All patients showed improved cognitive function after FMT compared with before FMT.	^272^
	a 90‐year‐old woman with Alzheimer's dementia	FMT from GI tract or other health problems, 27, male, and there is no use of drugs and antibiotics.	The patient's cognitive impairment improved.	^273^
PD	MPTP‐induced PD mice	FMT from PD group and healthy human controls group	FMT from healthy human controls can correct gut dysbacteriosis and ameliorate neurodegeneration by suppressing microgliosis and astrogliosis.	^274^
	Rotenone‐induced PD mice	Stools were collected from the control group mice delivered to recipient mouse via oral gavage	FMT treatment restored the gut microbial community, thus ameliorating the gastrointestinal dysfunctions and the motor deficits.	^95^
	PD patients with constipation	Frozen fecal microbiota	Patients who receive FMT experience an increase in gut microbiota abundance and relief from gait difficulties and constipation.	^275^
ASD	Propionic acid (PPA) rats model of autism	Feces from healthy donor rats	FMT restored PPA inducing ecological imbalance.	^276^
	ASD‐diagnosed children	standardized human gut microbiota that is >99% bacteria	ASD symptoms improved significantly and remained improved up to 8 weeks after the end of treatment, with an increase in overall bacterial diversity and abundance in the Bifidobacterium, Prevotella, and desulfonia taxa.	^277^
Depression	Fawn‐hooded (FH) rats	FMT from healthy rats model to FH rats	The gut microbiome could regulate the recipient's neurobiology and behavior via the systematic alternation of the depressive gut microbiota followed by the serum and hippocampal metabolism	^278^

Abbreviations: AP‐32, *Lactobacillus salivarius* subsp. salicinius AP‐32; Bf‐688, *Bifidobacterium animalis* subsp. lactis A6, BAA6; Bifidobacterium bifidum; CUMS, chronic unpredictable mild stress; FH, fawn‐hooded; FMT, fecal microbiota transplantation; FOS, fructo‐oligosaccharides; GOS, galacto‐oligosaccharides; LPN‐1, *Lactiplantibacillus plantarum* N‐1; LcS, *Lacticaseibacillus paracasei* strain Shirota; MMD, major depressive disorder; MOS, Mannan oligosaccharide; PP, *Pediococcus pentosaceus*; PPA, propionic acid; RM, residual medium; RS, resistant starch; SDS, social defeat stress; SHRs, spontaneously hypertensive rats.

### 5.1 Probiotics and prebiotics

In the past decade, the International Scientific Society for Probiotics and Prebiotics (ISAPP) has successively discussed and defined probiotics, prebiotics, metabiotics, and synbiotics.^279^, ^280^, ^281^, ^282^ Probiotics are “living microorganisms that, when consumed in sufficient amounts, confer health benefits to the host” (excluding undefined FMT).^279^ Prebiotics are health‐promoting substrates that are selectively utilized by host microbes and promote the proliferation of beneficial bacteria (*Bifidobacterium* and *Lactobacillus*).^280^ As described in Section 4, gut microbiome dysbiosis is common in neurological diseases, characterized by the depletion of beneficial and anti‐inflammatory bacteria. In most studies of probiotics related to neurological disorders, *Bifidobacterium* and *Lactobacillus* are the most studied probiotics, including the symptoms of ASD, ADHD, AD, PD, depression, and anxiety. After the use of *Bifidobacterium* or *Lactobacillus*, the condition of patients has been improved to varying degrees.^246^, ^259^, ^283^, ^284^, ^285^ It also suggests the potential of probiotic supplementation to modulate the gut microbiome for ameliorating neurological disorders. On the other hand, the mechanism by which probiotics improve neurological disorders has been explored in animal studies. For example, *Lactobacillus plantarum* MA2 attenuated neuronal degeneration and Aβ accumulation in the brain of rats with cognitive deficit and anxiety‐like behavior and alleviated intestinal mucosal injury and hindered microglia activation and neuroinflammation through TLR4/MYD88/NLRP3 signaling pathway.^286^ In addition, *L. plantarum* DP189 (DP189) inhibited tau hyperphosphorylation by regulating the PI3K/Akt/GSK‐3β pathway.^287^ In addition to the suppression of AD pathological states, in another study, DP189 also delayed the accumulation of αSyn in the substantia nigra of PD mice by inhibiting oxidative stress and proinflammatory responses, and increased probiotic abundance to reshape the gut microbiota of PD mice.^252^

The most widely demonstrated dietary prebiotics for human health benefit are the nondigestible fructo‐oligosaccharides (FOS) and galacto‐oligosaccharides (GOS). FOS and GOS reduced inflammation‐related anxiety and suppressed the increase in IL‐1β.^288^ Furthermore, following the administration of GOS to children with ASD, there was a marked improvement in social behavior scores and an increase in butyrate production in these children.^145^ The administration of FOS can improve cognitive impairments and pathological alterations in AD mice, as well as restore the disrupted microbial composition.^267^ Other prebiotic components can also offer positive impacts on neurological disorders. For instance, mannan can profoundly reshape the gut microbiome of AD mice, inhibit neuroinflammation and oxidative stress in the CNS of AD mice, and alleviate cognitive deficits and anxiety‐like behaviors.^266^ Resistant starch can alter the symptom severity of PD by modulating fecal SCFA levels.^268^ On the other hand, a synergistic combination of suitable probiotic and prebiotic compositions seems to have a more positive effect than either probiotic or prebiotic alone. For example, synbiotics 2000 mentioned in Section 4.1.2 shows the beneficial effects of synergistic combination of probiotics and prebiotics in the treatment of ADHD.^167^ Polymanuronic acid (PM) in combination with Lactobacillus rhamnosus GG (LGG) demonstrated superior neuroprotective effects compared with PM or LGG individually.^289^ However, we need to explore and study more abundant and effective combinations of specific probiotics and prebiotics.

### 5.2 Fecal microbiota transplantation

Fecal microbiota transplantation (FMT) is the introduction of gut microbiota obtained from the feces of healthy donors into the GI tract of patients to affect the symptoms or progression of neurological disorders through gut microbiota‐mediated immune, endocrine, metabolic and or neural pathways.^290^ Restoring the gut microbiome using FMT is now an accepted strategy.^291^ Preliminary literature suggests that FMT may be a promising therapeutic option for several neurological disorders.^292^ For example, FMT ameliorated depression‐like behaviors, altered the imbalance of gut microbiota, and attenuated mild stress‐induced intestinal inflammation, intestinal mucosal destruction, and neuroinflammation in rats.^293^ FMT has also shown a role in the treatment of depression in elderly patients.^294^ In several animal studies, FMT treatment improved motor deficits and GI disorders in PD mice and corrected gut microbial dysbiosis in PD mice.^95^ Meanwhile, FMT treatment has been used in PD patients with GI dysfunction in clinical trials and has reshaped the gut microbiota and improved motor deficits in PD patients.^275^ In another clinical trial of ASD, FMT improved GI and behavioral symptoms in ASD patients.^295^ However, neurological disorders often involve cognition, motor, inflammatory, and GI functions and even systemic responses. The combination of genetic and environmental factors is also mostly related to the pathogenesis, and the gut microbiota as a potential factor may affect the level of intestinal and systemic inflammation. In summary, clinical and preclinical trials of FMT for neurological disorders are still being further carried out to evaluate the efficacy and safety of FMT.

A prospective real‐world safety data from Hong Kong showed a favorable long‐term safety profile of FMT with a low risk of adverse events at 12 months after treatment.^296^ FMT is a kind of biological drugs, however, is not no adverse reactions. It has been reported that the common adverse reactions of FMT are usually GI discomfort, mainly including nausea, abdominal discomfort, abdominal colic, constipation, and abdominal distension.^272^, ^297^ These side effects are a natural response of the microbiota to the introduction of live microorganisms and their metabolites. Fortunately, they typically resolve quickly and do not pose significant health risks to the patient.^290^ However, the United States Food and Drug Administration has recently issued several FMT warnings following serious infections and one death following FMT.^291^ It also indicates the necessity of a safer and well‐designed donor microbiome for the implementation of FMT.

### 5.3 Dietary interventions

The impact of diet on gut microbiota has been succinctly discussed before (Section 2.2.1). Thus, maintaining a healthy diet is vital for managing gut microbiota, and dietary intervention therapy is an important treatment approach to consider. A dietary pattern rich in plants, limited in meat, low in fat, high in fiber, and abundant in antioxidants is linked to a decreased risk of advancing neurological disorders such as AD, PD, stroke, migraine, among others.^298^ There is a connection between diet and inflammation. In a healthy diet, fruits, vegetables, nuts, herbs, spices, and legumes serve as sources of anti‐inflammatory elements like omega‐3 fatty acids, polyphenols, vitamins, essential minerals, and probiotics, fostering a favorable brain environment.^299^ Conversely, a diet rich in processed foods, high‐fat items, refined carbohydrates, and red meat is linked to heightened inflammation and a greater susceptibility to neurological disorders.^299^ The healthy MD approach can also serve as a form of dietary intervention, particularly in promoting beneficial taxa and reducing inflammatory markers.^300^ The majority of evidences suggest that the MD has a protective effect on cognitive function, but there are some inconsistencies between studies and within.^301^, ^302^ The MD incorporates abundant anti‐inflammatory elements, suggesting that the positive impacts on cognitive function may stem from the synergistic or individual effects of various nutrients within the diet.^303^

The ketogenic diet (KD) is a low‐carbohydrate, high‐fat plan that induces significant changes in host metabolism by simulating fasting and encouraging the production and utilization of ketone bodies.^304^ The KD exerts neuroprotective effects in the CNS by suppressing glycolysis and promoting ketone body production. In a 4‐month intervention study with AD mice, spatial learning and memory abilities were enhanced, and neuroinflammation was diminished.^305^ However, the positive effect of KD on cognitive function depends on the initiation and duration of diet, with a shorter duration of KD treatment (2 months) having a weaker effect, and KD treatment starting in the late stage of AD (9 months) having no effect on cognitive improvement.^305^ Treatment with KD in PD mice ameliorated dopamine loss, inflammation, and motor dysfunction.^306^ Dysregulation of the gut microbiome in ASD mice can be reshaped by KD therapy.^307^ In addition to the beneficial results of the KD diet in animal studies, similar insights have emerged from the application of the KD model in human populations. Due to high fat content and drastic changes in dietary habits, classical KD (dominated by long‐chain triglycerides) is difficult to maintain for a long time, and to improve fitness, it is preferred to be based on medium‐chain triglycerides (MCT). In contrast, the population of AD patients treated with MCT showed improvement in cognitive function.^308^, ^309^ A total of five PD patients adhered to KD treatment for 28 days, and all showed improvement in UPDRS scores.^310^ However, the kinetic characteristics of ketone bodies appear to be strongly influenced by the formulation and dose of KD therapy. During starvation or KD treatment, ketone body concentrations in the brain and CSF are much lower than those in the blood.^311^ In addition, KD may also cause adverse reactions such as dehydration, hypoglycemia, hyponatremia, and dyslipidemia.^311^, ^312^ This also suggests that a standardized treatment regimen for folded KD needs to be established.

KD mimics the fasting state, so what is the effect of intermittent fasting (IF) on neurological disorders? It has been found that IF can alleviate LPS‐induced brain oxidative stress and cognitive impairment in animal models of systemic inflammation.^313^ Fasting mimicking diet (FMD) can prevent motor dysfunction in PD mice, and the gut microbiota may contribute to the neuroprotection of FMD in PD mice.^314^ Overactivation of the mechanistic target of rapamycin (mTOR) is associated with synaptic abnormalities of dendritic proteins and impaired autophagy and plays a role in ASD models and metabolism.^315^, ^316^ IF can enhance GABA tone, reduce mTOR activity, improve cognition and relieve anxiety, which may be another nonpharmacological intervention to improve the symptoms of AD and ASD.^315^, ^317^ However, more studies are needed to further elaborate the potential of dietary interventions in preventing the risk of neurological disorders.

## 6 CHALLENGES AND FUTURE DIRECTIONS

### 6.1 Limitations of current research

The development of gut microbiota is rapidly evolving, and the number of diseases associated with gut microbiota is increasing. However, the GBA is still in its early stage of research. As mentioned above, although there are many related researches, some research results are inconsistent or even opposite. In drawing on this information and in conducting research, caution is needed to avoid overinterpretation of these data. One problem with interpreting these findings, however, is that the studies were poorly powered or had false positives or negatives. Concerning probiotics, prebiotics, synbiotics, and other dietary or nutritional interventions, elucidating the rationale behind the selection, dosage, and route of administration of these nutritious dietary components is crucial. However, probiotics exhibit significant variability in stability and authenticity. These nutritional dietary components differ or target different microbial populations in the gut or system, which also means that some components are better suited to answering specific scientific questions. Moreover, it is crucial to examine whether these dietary components can be absorbed into the bloodstream and cross the BBB. This aspect requires further investigation, particularly in animal models. Notably, the beneficial effects of these nutrients are short‐lived, as probiotics typically fail to colonize the acidic gut environment for extended durations. Although probiotics offer therapeutic benefits, short‐term dietary interventions do not induce permanent changes in the gut environment, necessitating long‐term or more frequent consumption. Furthermore, variations in disease severity and duration also play a significant role in modulating gut microbiome alterations. Further research is needed to identify the target population for microbial therapeutic intervention, including determining the optimal disease stage and the age range of the patients.

Conversely, it is essential to take into account the distinctions between animals and humans when extrapolating the findings of animal experiments to humans. The data available on the translational applicability in rodents are quite restricted. One limitation of reporting animal studies is that there are few details about experimental design and execution and results.^318^ Therefore, the establishment of standardized and reproducible protocols would be beneficial in mitigating at least one source of variability.^318^ Moreover, there is a scarcity of animal models capable of replicating both peripheral and central pathology, highlighting the necessity for the creation of animal models that can emulate various disease stages, including prodromal phases like intestinal dysfunction prior to the onset of dyskinesia in PD patients.^207^ These diverse animal models could offer fresh perspectives on the gut microbiome.

Dietary interventions prescribed varied significantly among studies, especially concerning specific food quantity recommendations, whereas other lifestyle factors also influenced changes in the gut microbiome and behavior. FMT has been extensively researched in both clinical and preclinical settings and could hold promise as a treatment for various neurological disorders. However, FMT still faces uncertainties and lacks substantial evidence. Due to the imprecision in defining favorable microbiota, it is imperative to validate its benefits and long‐term effects, particularly regarding its potential to transfer immunomodulation and propagate pathogenic bacteria. Furthermore, there lacks a standardized protocol for fundamental procedures such as donor and recipient preparation for FMT, administration method and duration, and dosage. The rigor and reproducibility of this intervention warrant further investigation. Nevertheless, FMT is not entirely risk‐free, and there are still significant side effects that cannot be overlooked. These include inaccuracies in analyzing fecal donor material and exacerbation of chronic diseases in recipients.^290^ There are other important limitations to FMT studies in humans and animals. These limitations frequently stem from intricate research designs and encompass variations in antibiotic usage, FMT techniques, donor selection, as well as inadequate long‐term monitoring or a suitable control group. Despite two decades of research on gut microbiota, the exploration of mechanisms and treatment of neurological disorders through gut microbiota remains in its infancy. This underscores the long journey still ahead in gut microbiome research.

### 6.2 Potential future research directions

Due to the heterogeneity, complexity and dynamic changes of gut microbiota, there are limitations in many related studies of intestinal microbiota. In the future, more experimental studies and high‐quality large‐sample clinical trials are needed. In order to develop new therapeutic strategies, we must determine the translatability of these findings and, second, avoid overinterpretation of the data. Gut microbiota interventions could impact the availability and effectiveness of drugs through direct or indirect interactions with drug metabolism or by modulating immune responses. Whether FMT alone is adequate as a therapy remains uncertain and is contingent upon the particular characteristics of the disease and the bacteria involved in its regulation. Despite its application in clinical practice and the interest of clinicians and researchers, our understanding of the precise mechanisms and pharmacology of FMT remains incomplete, necessitating significant efforts to elucidate these mechanisms and continually update FMT guidelines worldwide. With the advancement of technology and the continuous development of artificial intelligence in the field of personalized medicine, the goal of scientific researchers, clinicians, and the community is to make FMT a standardized treatment approach in which the application of well‐defined microbial coalitions, metabolites, or laborator‐synthesized, easy‐to‐administer compounds to FMT is one of the current options.^291^

However, due to the absence of a double‐blind design in FMT studies and the potential influence of placebo effects, some studies may produce spurious results. This underscores the necessity for more extensive and larger double‐blind controlled trials. Furthermore, the potential benefits of FMT must be cautiously balanced against its potential risks. Safety should be the primary focus, with efficacy should considered a secondary endpoint. It has been argued that, in the future, capsules containing a coalition of beneficial bacteria may replace FMT, thereby increasing patient comfort and reducing potential side effects associated with the route of administration. Equally important, more evidence is needed on the amount of FMT required for each neurological disorder, and functional analyses of gut microbiota are needed to determine which key taxa play a key role in neurological disorders.

There is also a need to promote consensus on the definition of prescriptions for dietary interventions, some key development elements to improve the accuracy of dietary scoring, and the adoption of standardized tools to assess and detect adherence to dietary interventions. Studies on dietary interventions should adopt higher quality research methods to limit potential bias. In fact, further research is also needed to understand the internal and external factors that gut microbes are subjected to in response to nutritional or dietary changes. This requires the use of not only 16S rRNA sequencing, but also metagenomic analysis and multiomics approaches to further understand the structure of microbiota, including viruses and fungi.

## 7 CONCLUSION

At present, numerous clinical and preclinical studies have provided increasing evidence of the interconnection between gut microbiota and the brain. It is certain that the role of the MGBA in the pathogenesis of neurological disorders cannot be ignored. The MGBA regulates host health through the nervous system, immune pathways, and metabolic pathways. The healthy GI tract in a homeostatic state has a normal and stable gut symbiotic microbiota. Gut microbial translocation, alterations in composition, as well as the release, circulation, and accumulation of harmful metabolites, all contribute to the onset and development of neurological disorders. The gut microbiota can not only affect the state of glial cells but also regulate the synthesis of various neurotransmitters, playing an important role in neurodegeneration and neuroinflammation in neurological disorders.

Gut microbes are similarly involved in nervous system development at the beginning of life and in aging‐induced frailty and barrier penetration. Gut inflammation and dysbiosis caused by changes in diet, drugs, age and lifestyle may cause dysfunction of the GBA, which can progress into chronic systemic inflammation. Such immune‐mediated changes, enhanced release of proinflammatory cytokines, metabolic disorders, and increased BBB and GI tract permeability could potentially induce and trigger neuroinflammation and eventually induce neurological disorders. On the other hand, gut microbes are also two‐sided, and the increase or appropriate concentration level of beneficial metabolites can prevent and inhibit systemic inflammation and neuroinflammation to a certain extent, repair barrier penetration, and delay the progression of the disease.

Although many fundamental questions about the MGBA have not been answered, the current evidence also points the way for future research. For example, it remains unclear to what extent microbial metabolites are influenced by neuronal and/or hormonal pathways, as well as how many metabolites directly impact the brain after crossing the BBB. The role of both the CNS and the peripheral nervous system (e.g., ENS) in the MGBA requires further investigation. These potential issues and limitations in research provide new perspectives for future studies, particularly in relation to neurobehavioral disorders, neurodevelopmental disorders, and neurodegenerative diseases. Whether through direct microbial translocation, microbial metabolism, or indirectly via the nervous system, immune pathways, or metabolic pathways, real‐time environmental information can be provided to the nervous system. Current evidence supports that changes in the microbiome with dietary intake and other environmental factors may influence neurodevelopment and healthy aging. The mechanisms by which different microbiota are involved in neuropathy provide another promising avenue in the diagnosis, treatment and prevention of neurological disorders. These findings suggest that diet and lifestyle can be used to avoid disease risk. Therefore, there are many potential mechanisms of gut microbes in the pathological development of neurological disorders. These suggest that, when considering the intervention and adjuvant treatment of the pathophysiology of neurological disorders by gut microbes, the complex network of the MGBA still needs further exploration to fully characterize the effect of gut microbes on neurological disorders.

## AUTHOR CONTRIBUTIONS

Guolin Hong and Haiyue Liu designed the outline of article and suggestions of revision. Yuanyuan Yang and Hongzhang He drafted the Part I and collected the related articles. Lingjun Cheng and drafted the Part II of manuscript. Mingming You drew figures in the article and drafted the abstract, as well as the Part III and Part IV. Nan Chen drafted the Part V, and compiled the table in the article. Yanhua Cai and Yating Liu drafted the Part VI and concluded. All authors read and approved the manuscript.

## CONFLICT OF INTEREST STATEMENT

There is no conflict of interest to declare.

## ETHICS STATEMENT

Not applicable.

## Data Availability

Not applicable.
